# Regarding Perspective Piece from July 2016 “What Do We Know About Chagas Disease in the United States?”

**DOI:** 10.4269/ajtmh.16-1014

**Published:** 2017-03-08

**Authors:** Kenrad E. Nelson, Paul M. Ness, David A. Leiby

**Affiliations:** Department of Epidemiology, Johns Hopkins University, Baltimore, Maryland. E-mail: kenelson@jhsph.edu; 2 Department of Pathology, Johns Hopkins School of Medicine, Baltimore, Maryland. E-mail: pness@jhmi.edu; 3 U.S. Food and Drug Administration, Silver Spring, Maryland. E-mail: david.leiby@fda.hhs.gov

Dear Sir:

We are interested in the Perspective Piece, “What do we know about Chagas disease in the United States?” written by Montgomery and others that was published recently in the Journal.^1^

A study of *Trypanosoma cruzi* prevalence and incidence that we performed in 2000 among 11,430 cardiac surgery patients in two large hospitals in Houston and Johns Hopkins Hospital in Baltimore provides additional data on the epidemiology of Chagas disease in the United States.^2^

This study was not cited in the perspective by Montgomery and others. We detected six patients (0.05%) who were repeatedly reactive by enzyme immunoassay and were subsequently confirmed as positive by radioimmunoprecipitation assay. All six had received blood transfusions. However, four of the six patients with available pretransfusion samples were positive prior to their surgery. Five of the six patients were Hispanic and one patient had never traveled outside of the United States. He was a consulting geologist who had a history of frequent field trips to rural areas of Texas where previous cases of autochthonous *T. cruzi* had been reported. Two of the patients in this study had undergone heart transplants, including the patient who had not traveled outside the United States. Preserved tissues from hearts removed at surgery were tested by polymerase chain reaction for the presence of parasite DNA, and in both cases were positive for *T. cruzi*. None of the medical providers, including their cardiologists, surgeons, and pathologists had considered the diagnosis of Chagas disease in any of these patients. Overall, 184 (1.8%) of the 12,219 patients in this study were Hispanic and 2.7% of these Hispanic patients were confirmed seropositive for *T. cruzi*.

Our study highlighted several features of Chagas disease in the United States. First, Chagas disease is often not included in the differential diagnosis of Hispanic cardiac patients among clinicians in the United States. Second, Chagas disease cardiomyopathy is not uncommon among immigrants from Latin American countries. Screening of blood donors has effectively prevented the transmission of *T. cruzi* by transfusion in the United States. However, screening of patients who have emigrated from areas endemic for Chagas disease, especially those with cardiac disease, is not often done, but should be emphasized.
